# Genetic instability as a driver for immune surveillance

**DOI:** 10.1186/s40425-019-0795-6

**Published:** 2019-12-11

**Authors:** Guim Aguadé-Gorgorió, Ricard Solé

**Affiliations:** 1 ICREA-Complex Systems Lab, Universitat Pompeu Fabra, Barcelona, 08003 Spain; 2 Institut de Biologia Evolutiva (CSIC-UPF), Psg Maritim Barceloneta, 37, Barcelona, 08003 Spain; 3 Santa Fe Institute, 399 Hyde Park Road, Santa Fe, 87501 NM USA

**Keywords:** Genetic instability, Neoantigen load, Mismatch repair, Immune surveillance, Combination therapies

## Abstract

***:**

BackgroundGenetic instability is known to relate with carcinogenesis by providing tumors with a mechanism for fast adaptation. However, mounting evidence also indicates causal relation between genetic instability and improved cancer prognosis resulting from efficient immune response. Highly unstable tumors seem to accumulate mutational burdens that result in dynamical landscapes of neoantigen production, eventually inducing acute immune recognition. How are tumor instability and enhanced immune response related? An important step towards future developments involving combined therapies would benefit from unraveling this connection.

***:**

MethodsIn this paper we present a minimal mathematical model to describe the ecological interactions that couple tumor adaptation and immune recognition while making use of available experimental estimates of relevant parameters. The possible evolutionary trade-offs associated to both cancer replication and T cell response are analysed, and the roles of mutational load and immune activation in governing prognosis are studied.

***:**

ResultsModeling and available data indicate that cancer-clearance states become attainable when both mutational load and immune migration are enhanced. Furthermore, the model predicts the presence of well-defined transitions towards tumor control and eradication after increases in genetic instability numerically consistent with recent experiments of tumor control after Mismatch Repair knockout in mice.

***:**

ConclusionsThese two main results indicate a potential role of genetic instability as a driver of transitions towards immune control of tumors, as well as the effectiveness of increasing mutational loads prior to adoptive cell therapies. This mathematical framework is therefore a quantitative step towards predicting the outcomes of combined therapies where genetic instability might play a key role.

## Background

Cancer is a disease resulting from Darwinian evolution in cellular tissues[1]. Following depletion of a vast set of genetic insults altering normal multicellularity phenotypes, rogue cells are able to adapt and evade selection barriers leading to uncontrolled proliferation. In this context, genomic instability plays a key role as a driver of the genetic novelties required for tumor progression and rapidly adapting phenotypes [2, 3]. High levels of evolving instability sustain a very diverse population [4], and intra-tumor heterogeneity lies at the very core of why cancer is still difficult to define, characterize and cure [5].

In this paper we aim at understanding an important relationship between the effectiveness of cancer immunotherapy and genetic instability. The relevance of such link needs to be found in the challenges faced by immunotherapies based on immune checkpoint inhibition or adoptive cell transfer [6], where mutational burden seems to play a key role. Due to the underlying complexity of cancer immunology, interdisciplinary efforts towards novel immunotherapies are much required [7–9]. As discussed below, the crucible of the problem might be to the nonlinear dynamics associated to cancer neoantigen production and the consequent enhancement of immune surveillance.

A key point in cancer immunotherapy lies on the mechanisms by which T cells actually recognize cancerous from healthy tissue [10] and eventually attack tumor cells expressing tumor-specific antigens [11]. On a general basis, such antigens can be common proteins for which T cell acceptance is incomplete, or more importantly, novel peptides [10, 12]. Except for specific tumor types of viral etiology, these so-called *neoantigens* arise after DNA damage resulting in the production of novel proteins. Recent advances highlight the importance of understanding neoantigen generation as a consequence of the tumor mutational load and dissecting specific neoantigen immunogeneicity [10, 11, 13]. Furthermore, direct correlations have been suggested between neoantigen production at high microsatellite instability, eventual immune surveillance and clinical response to immunotherapies [14–16].

Several experimental and clinical sources are pointing towards a causal relation, including tumor growth impairment after inactivation of MLH1 [17], or the positive response to PD-1 blockade across different mismatch repair (MMR) defficient cancer types [18]. The inactivation of MMR results in increased mutational burden of cancer cells, promoting the generation of neoantigens which improve immune surveillance and eventual tumor arrest. These obxservations suggest a novel view on immunotherapy, where targeting mutagenic pathways can result in an alternative mechanism to unleash immune responses [9, 19].

All in all, genetic instability seems to play a conflictive role in cancer evolution and proliferation. It appears that the same genome alterations that activate cancer progression can trigger T cell recognition and immune attack. The extent of such trade-off and its application to therapy, however, is not clear. On the one hand, mutagenic therapies coexist with an intrisic risk, as increased genetic instability on heterogeneous populations might activate oncogenic outgrowth in previously stable cells. Moreover, a reactive immune system might pose a selective pressure for immune editing, leading to selection for T cell evading tumor subclones. How do these two components -instability and immune response- interact and what are the consequences? Is it possible to provide useful insight from mathematical models without a detailed picture of the immune landscape of cancer?.

Nonlinear responses associated to cancer-immune system interactions have been known from the early days of cancer modelling, from more classical approaches [20] to recent perspectives based on neoantigen recognition fitness [21]. These studies have revealed a number of interesting properties exhibited by toy models, including in particular the existence of shifts and breakpoints separating cancer progression from its extinction (see [22] and references therein). Such shifts are of exceptional importance in our context: they indicate the existence of well defined conditions (and perhaps therapeutic strategies) allowing an all-or-none response. However, a mathematical description of the specific role of genetic instability in cancer immunology has not yet been developed. Below we provide a first approach to such goal, based on considering both cancer adaptation and immune surveillance as influenced by mutational burden, and we analyze how genetic instability can account for transitions towards states of cancer control and elimination. The implications of these transitions on combination therapies are discussed, pointing towards possible cross-therapies activating neoantigen production and immune stimulation.

## Methods

### Population dynamics of the tumor-immune interaction

The ecology of the cancer-immune system interaction pervades several complexity levels, from a vast antigenome [23] to multilayer cellular competition dynamics [24], and a first step towards modeling such ecology lies in dissecting which specific ingredients are key drivers in the phenomena we aim to understand.

Recent research points out that there might be up to 28 immune cell types with both antitumor and immunosupressive roles infiltrated within a tumor [25]. Focusing on the immuno-surveillance mechanism of tumor growth inhibition following immune system recognition (early introduced in [26]), a minimal modelling approach recalls at least considering a population of tumor cells growing in competition with immune cells. It is commonly accepted that the immune response to cancer is mostly driven by an adaptive cohort of *cytotoxic immune cells*, such as CD8 ^+^ T cells, together with a cellular compartment of the *innate* immune system such as NK cells [27, 28]. Despite this work focuses on the adaptive response to neoantigen presentation, including an innate effector response will allow for understanding relevant non-antigenic immune effects.

Even if further models have been useful at depicting very advanced properties of the immune system [29], we have chosen to keep a minimal scenario able to describe the competition dynamics at play. We apply a well characterised model (see e.g. [30]) that has been used to account for experimental results in cancer immunology such as tumor-immune equilibrium [31]. This model has been studied using parameter ranges measured from experimental setups consistent with several tumor types (Table 1, see [20, 32]).

**Table 1:** Parameter values for the cancer-immune ecology model, estimated from experimental data of BCL _1_ lymphoma in the spleen of chimeric mice (see [20])

Parameter	Meaning	Kuznetsov et al. (1994) estimate
r	Cancer cell replication rate	0.18day ^−1^
K	Tumor carrying capacity	2×10^9^cells
*δ*_*c*_	Immune-mediated cancer cell death rate	1.101×10^−7^*day*^−1^*cells*^−1^
*δ*_*E*_	Cancer-mediated immune cell death rate	3.422×10^−10^*day*^−1^*cells*^−1^
m	Rate of T cell migration towards tumor site	1.3×10^4^cells day ^−1^
g	Tumor size limit for effective T cell infiltration	2.019×10^7^cells
*ρ*	Rate of cancer cell recognition by the immune system	0.1245day ^−1^
d	Intrinsic T cell death rate	0.0412day ^−1^

The cellular interactions considered here involve a commonly used well-mixed (mean-field) model [20, 22] where the population of cancer cells *c* follows a logistic growth (at effective replication rate *r*=*b*−*d* and carrying capacity *K*) and immune-cell mediated death (at rate *δ*_*c*_). This saturating growth model captures several tumor microenviroment effects of malignant cell competition and death, such as spatial constraints or nutrient availability [33].

1 $$ \frac{dc}{dt} = rc \left(1-{c \over K} \right) - \delta_{c}c E.  $$

The effector immune population includes both NK and T cell compartments. Despite further modeling has been able to capture specific dynamics of T cell activation by cancer-NK cell encounter [27], activation of both cell types by malignancy can be described in a similar form [22], here described by

2 $$ \frac{dE}{dt} = m + \rho \left({c \over g+c} \right)E - \delta_{E} c E - d E,  $$

**Fig. 1:**
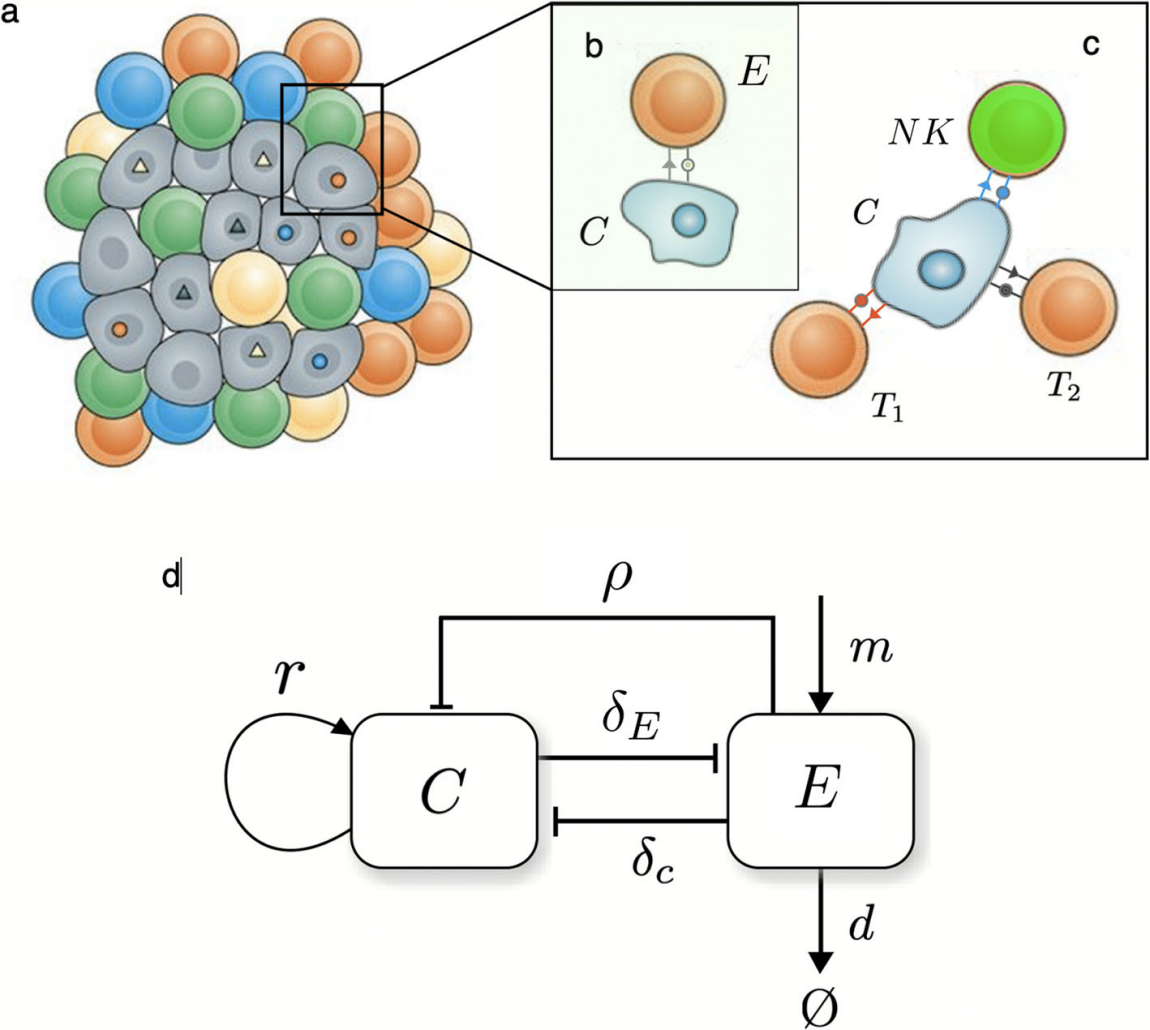
A schematic summary of the basic cancer-immune cell-cell interactions. The two key components are (**a**) a tumor population driven by genetic instability and (**b**–**c**) interactions associated to tumor cell recognition and attack by T and NK cells. The strength T cell attack depends on the number of surface neoantigens (**c**), while NK killing is constant [27]. In (**d**) the population-level interaction diagram is displayed based on the model in [20]. Here *c* and *E* indicate the number of cancer and T and NK cells, respectively. Cancer cells grow at a rate *r* (and have a limited carrying capacity) while immune cells enter the system at a constant production rate *m* and react at malignant cells at a rate *ρ* that will be different for NK cells and instability-dependent T cell recognition. A constant average death rate *d* is associated with their removal. Two constant cross-interactions rates are also indicated as *δ*_*T*_ and *δ*_*c*_ associated to the removal efficiency of cancer cells and the death of immune cells resulting from the same process, respectively

In this framework, the innate and adaptive immune populations are encapsulated into a single Effector compartment that grows due to a constant migration of cells and a predation term *ρ* that is commonly acknowledged to obey a Michaelis-Menten-like saturation due to limitations in immune cell circulation through the tissue [20] and penetration within the solid tumor [32, 34]. The peculiarity of the model lies in considering this predation term different for both NK and T cells. As discussed below, *ρ* is split into a constant rate refering to innate NK predation (see [27] and references therein) together with a variable part that will relate to antigen recognition by T cells, so that *ρ*=*ρ*_*NK*_+*ρ*_*T*_. Effector cells also have a natural decay rate, *d*, and die when competing with tumor cells at a rate −*δ*_*E*_*c*. The complete set of interactions described by (1) and (2) is schematically shown in Fig. 1.

### Ecological trade-offs in genetic instability

As discussed above, genetic instability plays a key role in tumor evolution, acting as the driving mechanism towards phenotypic variation and adaptation. Within our model, this can be translated as the replication rate being a function of its level of genetic instability *μ*. On the other hand *ρ*_*T*_, the rate of cancer cell recognition by T cells, is also *μ*-dependent because of neoantigen production. Below we propose a minimal characterization of *r* and *ρ* able to describe how genetic instability modulates such trade-off.

#### Cancer adaptation as a function of genetic instability

Cancer adaptation, here summarized to modulations in its replication rate, stems from the phenotypic plasticity resulting from mutations and copy-number alterations. On a general basis, enhanced tumor replication follows from mutations affecting oncogenic pathways, which poses a trade-off on genetic instability as it can, as well, damage any of the necessary machinery for cell viability.

Following previous research [35, 36], an adaptive landscape is build on several assumptions based on the probabilities of mutating oncogenic and house-keeping genes.

Genetic instability has a twofold impact on cell fitness. Specifically, replication rate *r* will be considered a function of mutation probability *μ*. A landscape *r*(*μ*) is now in place [35, 37], and follows from considering that mutations on oncogenes can translate into a linear increase in replication rate. This follows from assuming that reproductive effects of oncogenes, as for advantageous mutations on many systems, are exponentially distributed [38], so that their sum is gamma distributed with average increasing with the number of mutated oncogenes. This will be expressed as *R*_1_(*μ*)=*r*_0_+*N*_*R*_*δ*_*R*_*μ* with *r*_0_ being the basal replication rate of normal cells, *N*_*R*_ the number of oncogenes responsible for increased replication and *δ*_*R*_ the mean effect on replication rate when mutating one of such genes.

To account for cell viability, the number of house-keeping genes *N*_*HK*_ is taken into account so that mutations affecting them result in null replication [39]. This introduces the constraint of not having any of them mutated, $\phantom {\dot {i}\!}R_{2}(\mu)=(1-\mu)^{N_{HK}}$. Grouping both considerations together we obtain an analytical description of the coupling between replication rate and mutation probability *r*(*μ*)=*R*_1_(*μ*)*R*_2_(*μ*) which reads:

3 $$ r(\mu) = (r_{0}+N_{R}\delta_{R}\mu)(1-\mu)^{N_{HK}}  $$

This adaptive landscape is of course of qualitative nature, and realistic fitness landscapes for unstable tumor environments are still far from our knowledge. However, certain points can be made if we give values within realistic parameter ranges to our function. The number of both oncogenes and house-keeping genes have been widely assessed, and we take them to be about *N*_*R*_≈140 [40] and *N*_*HK*_≈3804 [39] respectively. Interestingly, considering small replication effects for *δ*_*R*_, such experimental values produce an adaptive landscape that has an optimal region for tumor replication at about *μ*≈10^−5^−10^−4^, which is in accordance with the point-mutation probability levels experimentally measured for unstable tumor cells [41].

#### Immune recognition of malignancy as a function of genetic instability

Building a mathematical description of how the immune system reacts at the mutational burden of cancer cells is not straightforward. This stems from the fact that such behavior is yet starting to be understood at the molecular level and it probably builds upon many layers of complexity [10]. In our minimal mathematical approach, the first step is describing immune reactivity as proportional to the adaptive compartment of cancer cell recognition *ρ*_*T*_, a rate that itself depends on the dynamics of neoantigen expression. Under our assumptions, since adaptive immune response follows from neoantigen detection we expect *ρ*_*T*_ being a function of the overall mutational landscape of a tumor, *μt*, which is eventually responsible for such neoantigen dynamics. Following recognition probability distributions from [21], we expect the average dominance to initially increase with mutations as more and more neoantigens are generated and eventually saturate as very dominant neoantigens are rare.

The mathematical shape of this dependency *ρ*_*T*_(*μt*) could stem from purely stochastic dynamics, but recent research gives better insight into the shape of this correlation. Rooney and colleagues provided an enlighting perspective in this direction by comparing a measure of immune response from the transcript levels of two key cytolytic effectors with the total mutation count for eight tumor types [42].

**Fig. 2:**
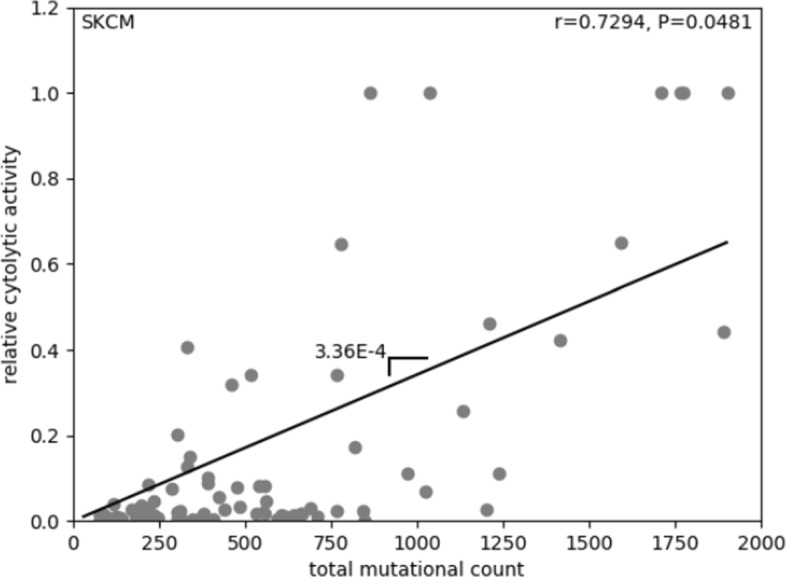
Measuring immune reactivity as a function of the mutational load. Melanoma is plotted as an example, where a linear regression (black line, scale=3.36E-4) between total mutation count and relative cytolytic activity is evaluated. Results for 12 cancer types in Table 2. Data is obtained from [42]. As in the original work, the correlation spans the 5 ^*th*^ to 95 ^*th*^ percentile of the mutation count variable

Cytolytic response strengths in [42] seem to indicate a dependency on tissue and tumor microenviroment, which we have not included in our study since our model is not tumor type-specific. For each tumor type, a least-squares linear regression is used (Melanoma in Fig. 2). When comparing across tumor types the shape of the immune response seems to obey a common pattern across many cancers, once cytolytic response values are normalized (Table 2). A linear relation can be found for which normalized cytolytic activity scales with mutational load as CYT ∼4.35×10^−4^*μt* when averaged across the range of tumor types explored here. However, we expect a function depending only on mutation probability. The variable *t* in this expression refers to the evolutionary life history of mutations accumulation of the tumor. This time scale is much larger than the faster ecological dynamics that govern the cancer-immune system interactions, so that we can consider it an average measure of tumor age at the time of detection, and consider it constant when introducing *ρ* in the ecological dynamics. From these facts, the only variable governing immune recognition at the cancer-immune competition level is the point mutation probability *μ*.

**Table 2:** Linear regressions for *ρ*(*μt*) across 12 cancer types, resulting in *ρ*(*μt*)=4.35×10^−4^*μt*

Cancer Type	Gradient of *ρ*(*μt*)
GBM	1.38 ×10^−4^
LUAD	4.73 ×10^−4^
LUSC	6.98 ×10^−4^
BRCA	2.17 ×10^−3^
UCEC	2.30 ×10^−4^
CRC	2.88 ×10^−4^
STAD	3.29 ×10^−4^
HNSC	8.37 ×10^−4^
SKCM	3.36 ×10^−4^
CESC	9.25 ×10^−4^
BLCA	3.98 ×10^−4^
LGG	2.98 ×10^−3^

Data is obtained from [42] with linear regressions performed as in Fig. 2

A very rough estimate for *t* could be either inferred from average cell replication data or from the fact that values for the mean mutation rate and the absolute mutational load are known for many tumors [43]. For example, we can use the notion that mutator tumors have mutation rates of about 10^−5^ mutations per gene per cell division [44], which account for the accumulation of about 10^3^ somatic mutations per tumor life [42], so that average tumor divisions lies at about *t*∼10^7^. Using this approximation we obtain our preliminar expression for how the immune reactivity rate depends on the mutation levels, *ρ*_*T*_(*μ*)=4.35×10^3^*μ*.

**Fig. 3:**
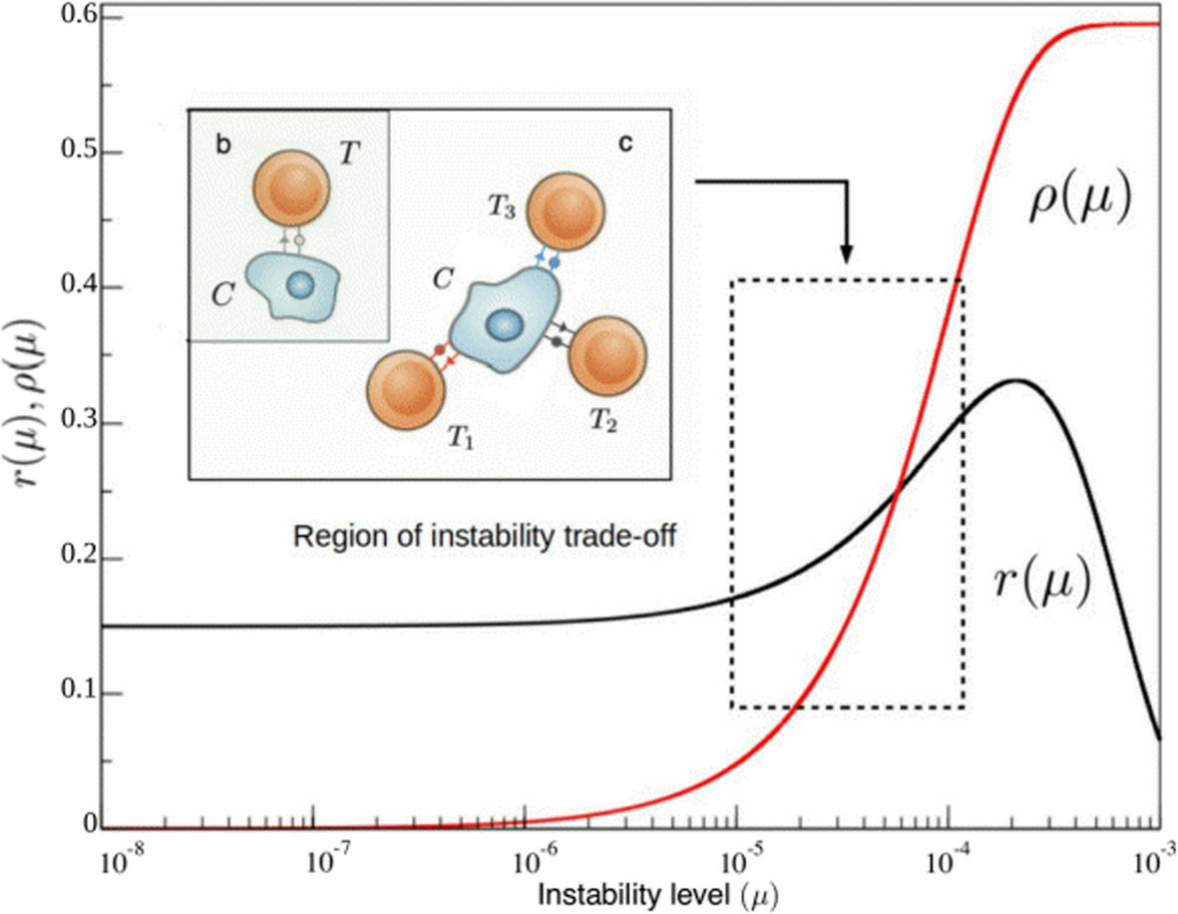
Functional forms for cancer replication *r*(*μ*) and the adaptive compartment of immune recognition *ρ*_*T*_(*μ*) related to neoantigen presentation. The first (black curve) provides a representation of the cancer instability landscape, as predicted from our theoretical approach (see Methods section) and calibrated by available data. It reveals a very slow increase (in this log-linear diagram) at low instability levels followed by an increase associated to favourable mutations allowing for faster replication and a marked decay at high instabilities due to mutations on viability genes. The immune reactivity to genetic instability function *ρ*(*μ*) (in red, obtained from [42]) rises from zero to saturation beyond *μ*∼10^−4^. The relevant domain of common cancer instability levels is highlighted. The innate response, *ρ*_*NK*_, is not depicted as is not a function of genetic instability and lies in a smaller order of magnitude of around *ρ*_*NK*_=2.5×10^−^2 [27]

In this first correlation measure from [42], however, immune recognition grows constantly with mutational load. This growth should not be indefinite, and many factors counteract the cytolytic effect of antigen-producing mutations. As an example, increases in genetic instability can also account for antigen silencing and immune editing, which itself would reduce cytolytic activity [45]. All in all, it seems plausible to consider that antigenic and immune-suppressing mutations could balance beyond certain mutational threshold. Following data from [42] it seems that the tumor-immune cytolytic interaction is far from saturation, with an estimated saturation behavior to happen beyond *μ*∼10^−4^, a mutational level higher than those of most tumors measured by recent methodologies (see e.g. [42]). This saturating function follows the same trend of the data-based linear relationship and reads

4 $$ \rho_{T}(\mu)=4.35\times 10^{3} \mu \sim \frac{2}{1.4 + e^{14000\mu}} - \frac{5}{6},  $$

and can be compared with tumor adaptability *r*(*μ*) (Fig. 3) to obtain a full mutational landscape for tumor progression in the presence of T cells. Assumptions on immune response saturation at high genetic instabilities do not affect the outcome of the model. Finally, the death rate of cancer cells increases as they become immunogenic and detectable by T cells [10, 46]. This is translated in the model as cancer cells dying at rate *δ*_*c*_=(*ρ*_*NK*_+*ρ*_*T*_(*μ*))*δ*, the rate of immune detection (*ρ*) times the rate of T cell killing (*δ*). Since saturating dynamics are already present in the mathematical shape of *ρ*_*T*_, this last rate *δ* is considered constant, which is consistent with other recent modeling efforts [46].

### Cancer-Immune system attractor states

Once the proper role of genetic instability on cancer adaptation and immune response is defined, the original model is reinterpreted as a pair of coupled populations with instability-dependent rates, i.e.

5 $$ {\frac{dc}{dt} = r(\mu)c\left(1-{c \over K}\right) - \delta(\rho_{NK} + \rho_{T}(\mu))c E}  $$

6 $$ {\frac{dE}{dt} = \frac{(\rho_{NK} + \rho_{T}(\mu))}{g+c}cE + m - \delta_{E} c E - d E }  $$

A global picture for the behavior of the system is obtained by studying its possible attractor states taking into account the variability of the mutational load. Together with the cancer free attractor (*c*^∗^,*E*^∗^)=(0,*m*/*d*), other attractors can be inferred from the intersections between nullclines

7 $$ \begin{aligned} E_{1}(c) &= \frac{r(\mu)}{\delta(\rho_{NK} + \rho_{T}(\mu))}\left(1-{c \over K}\right) \\ E_{2}(c) &= \frac{m}{\left(\delta_{E}c + d - \frac{(\rho_{NK} + \rho_{T}(\mu)) c}{g + c} \right)} \end{aligned}  $$

**Fig. 4:**
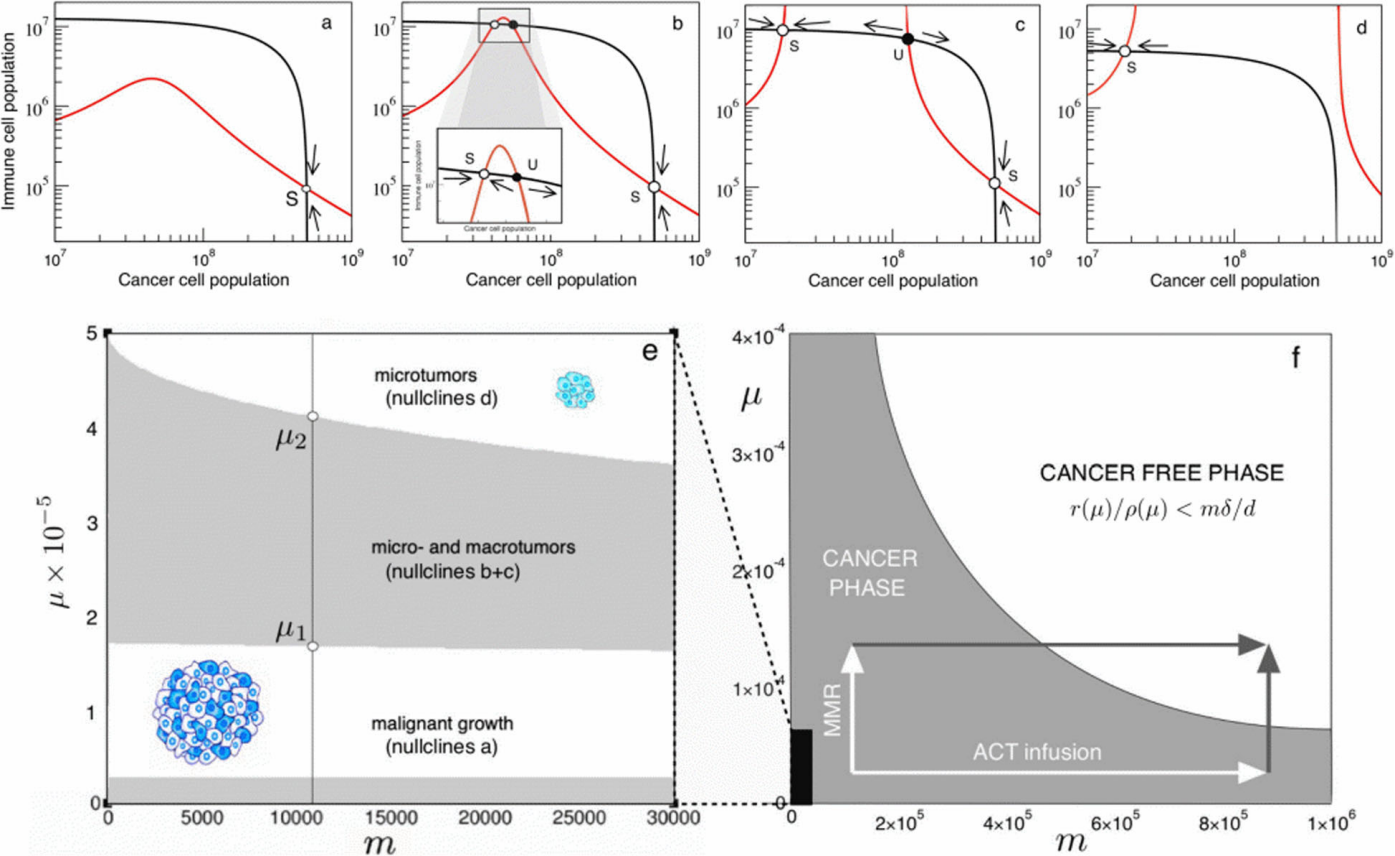
Cancer-Immune response attractors driven by instability. In (**a**–**d**) we display the nullclines as we increase mutation probability values. Arrows indicate the system flow towards the small and large tumor attractors. Two transitions can be seen. **a** At low genetic instability levels of 10 ^−5^ mutations per gene per division, such as those common in mutator tumors, only a large cancer attractor coexists with the unstable tumor-free equilibrium left from the graph at *c*=0. **b** Beyond *μ*^∗^∼1.6×10^−5^, two new attractors are created, which correspond to a stable microtumor attractor and an unstable twin [30]. **c** At *μ*^∗^=2.0×10^−5^, the microtumor attractor becomes smaller; until eventually the attractor of uncontrolled tumor growth is eliminated (**d**) at mutational levels similar to those attained after Mismatch-Repair knockout [40]. In (**e**) and (**f**) we summarise the bifurcation diagrams for the possible scenarios as a function of *μ* and *m*. For standard immue migration rates (**e**, black region in **f**), mutational increases drive the system across the two transitions observed in (**a**–**d**) and towards the controlled tumor state. However, by increasing both *μ* and *m* through combining Mismatch Repair knockout with adoptive cell therapy, the total cancer clearance state can be accessed

Nullcline 1 is a simple line with a negative slope controlled by the inverse of the carrying capacity of cancer cells. On the other hand, nullcline 2 is a peaked curve, with a height controlled by immune cell migration and a denominator that might eventually produce divergences. Through their crossings we will find which steady states coexist under which parameter domains (See Results section and Fig. 4).

Along with genetic instability, another parameter is key to the dynamics of the system. Regarding the second nullcline, we can see its size is linearly affected by the influx *m* of immune cells arriving at the tumor site. It is therefore interesting to understand how *μ* and *m* are related to cancer-immune scenarios, since this will open the door to further discussion on mutagenic and immune activation therapies.

By solving *E*_1_(*c*)=*E*_2_(*c*), we can understand how the values of *m* and *μ* affect the nature and number of possible solutions of the system. We here write (*ρ*_*NK*_+*ρ*_*T*_(*μ*))=*ρ* for simplicity. The previous identity leads to a cubic expression of the form *Ac*^3^+*Bc*^2^+*Cc*+*D*=0, with

8 $$ \begin{aligned} A & = -\frac{\delta_{E}r(\mu) b}{\delta\rho} \\ B & = -\frac{r(\mu) }{\delta\rho} \Big(b(d-\rho) + \delta_{E}(bg-1) \Big) \\ C & = \frac{r(\mu) }{\delta\rho} \Big(d + g\delta_{E} - \rho - bgd \Big) - m \\ D& = \frac{r(\mu) g d }{\delta\rho} - mg. \end{aligned}  $$

The sign of the discrimant *Δ*=18*ABCD*−4*B*^3^*D*+*B*^2^*C*^2^−4*AC*^3^−27*A*^2^*D*^2^ will define of which combinations of *m* and *μ* belong to which scenarios of Fig. 4. Knowing that three real roots exists for *Δ*>0 and only one for *Δ*<0, the transitions between attractor scenarios happen to occur at *Δ*=0. This condition can be used to easily describe the whole bifurcation space as seen in the results and Fig. 4e and f, showing how mutation frequencies and immune stimulation affect the possible outcomes of the system.

## Results

### Minimal mutation rate for an efficient immune response

Before engaging into a full analysis of the complete model, we can study the behavior of the system for initial phases of progression. This corresponds to a small tumor of size *c*<<*K*=2×10^9^ cells. Under this assumption, the population dynamics of *c*(*t*) simplifies to

9 $$ \displaystyle{\frac{dc}{dt} = c\Big(r(\mu) - \delta(\rho_{NK} + \rho_{T}(\mu)) E - d_{c}}\Big)  $$

where we have now included a natural death rate −*d*_*c*_ that accounts for growth barriers of initial malignant cells if away from the microenviroment carrying capacity [33]. From (9) we can isolate a condition for tumor control, i.e.:

10 $$ \frac{dc}{dt} < 0  $$

which leads to a crude estimate of the amount of effector immune cells required to counterbalance tumor growth, namely

11 $$ E(\mu)>\frac{r(\mu)-d_{c}}{\delta_{C}(\rho_{NK} + \rho_{T}(\mu))}.  $$

The inequality consistently shows that *E*(*μ*) will be proportional to the instability landscape of cancer growth rate divided by both NK and immune-mediated death. This acknowledges that both NK or T cells can play crucial roles in cancer surveillance. To understand the role of the adaptive compartment and genetic instability in controling a growing cancer population, we use validated data from [20] (Table 1) and consider a healthy adaptive immune population of *T*∼10^7^ cells ([29] and following sections), to obtain that the immune control condition is fulfilled for *μ*>5.75×10^−5^ mutations per gene and replication. This can be understood as the minimal mutation rate required to generate a critical neoantigen load for T-cell immune attack, not considering here NK or other innate components away from the scope of the work. The estimated value is consistend within the range of genetic instability levels associated to MMR knockout [47], indicating a connection between mutagenic therapies enhancing genetic instability and a threshold level to activate the immune response.

### Transitions to tumor control and eradication at genetic instabilities within the mMR-knockout range

For well-formed tumors, no similar approach can be performed, but we can study the effects of changes in genetic instability in the sytem defined by equations (4) and (5) by picturing the intersections between nullclines described in the Methods section. As we are interested in the specific role of genetic instability and neoantigen presentation, we will focus here on the adaptive part of immune recognition, *ρ*(*μ*). It is straightforward to see how several transitions regarding creation and anihilation of steady states are governed by mutational probability *μ* (Fig. 4a-d).

As expected from [30] and previous discussions, we know that the cancer-free attractor will always be present, but local stability will be ensured if *r*(*μ*)/(*ρ*_*NK*_+*ρ*_*T*_(*μ*))<*mδ*/*d* (depicted in Fig. 4f). Without an innate component, the condition is only fulfilled at very high instability levels above 10 ^−4^ mutations per gene per division. This implies that no complete tumor clearance solely by neoantigen recognition seems possible at realistic mutation rates for fixed *m*, meaning that an innate response might also play a role in complete respondant patients, as many therapies do elicit total tumor eradication [45]. Additionally, we can see that a large-tumor solution *c*_*L*_ is also present at low instabilities (Fig. 4a), and it is globally asymptotically stable. Interestingly, a transition seems to occur as the value for *μ* becomes larger: before *E*_2_(*c*) diverges, a smaller stable attractor *c*_*S*_ is created together with its unstable *twin* (Fig. 4b), which is often described as a microtumor controlled by the immune system. Furthermore, nullcline 2 diverges at *μ*∼1.75×10^−5^ (Fig. 4c), and, as the two values for divergence of *E*_2_(*c*) grow further appart, the large cancer attractor disappears and only the controlled microtumor coexists with the cancer free attractor and is globally asymptotically stable (Fig. 4d). These results are consistent with those of [30], where such solution is considered a microtumor controlled by the immune system. However, both transitions of microtumor creation and large tumor elimination being a function of the mutational levels of the tumor population are new to the present work.

At this point it is clear that understanding at what instability levels these transitions happen is key to the possible outcomes of the tumor-immune interaction. For the given parameter region and in the absence of a strong innate response, a basic computational approach lets us see that the first transition happens around *μ*∼1.65×10^−5^ (Fig. 4b), whereas another transition where the large tumor attractor disappears happens at higher *μ* values of about *μ*∼4×10^−5^ (Fig. 4d).

Following extensive data, unleashed genetic instability after Mlh1 knockout in mice accounts for increasing mutation frequencies ranging from 10 ^−6^∼10^−5^ up to 10 ^−4^ mutations per gene per division (values assessed for transgenic mice containing *supFG1* or *cII* from [47]). Interestingly, instability levels before MMR knockout put our system within a region where the large cancer attractor is stable and no controlled microtumor exists. However, the increase after Mlh1 knockout might be pushing cancer cells to a region beyond $\mu ^{*}_{1}$, where the stable microtumor attractor appears, or even $\mu ^{*}_{2}$, where the stable large cancer attractor has disappeared (Fig. 4e).

The resemblance between the model and experiments linking genetic instability to adaptive immune surveillance seems intuitive enough. Following [17], we think that there is a connection between the observed phenomenon of immune reactivity and tumor collapse after Mismatch Repair knockout and the qualitative behavior of our model, which depicts a transition of this kind at high *μ* values. Furthermore, we have taken advantage of recent research in order to use quantitative data to build our model. The fact that our model predicts the range for which immune surveillance reacts at increased cancer instability levels emphasizes the possible existence of transitions like the ones studied here.

Assessing if these two transitions are in fact well defined in vitro or if genetic instability can modulate tumor evolution towards controlled states can shed new light into the precise nature of mutagenic therapy as a mechanism towards increasing tumor immunogeneicity. Such therapies have produced key results in the field of virology [48], but, within the context of cancer, recent insight seems to indicate that increasing the immunogeneicity of a tumor preludes evolution of subclonal neoantigen heterogeneity [49–51].

### Implications on immune surveillance: the role of tumor size

Besides the possible implications for mutagenic therapy as a facilitator of immunotherapy effectiveness, the fact that genetic instability shapes the landscape of the cancer-immune interaction has further implications on the fate of tumor growth. Tumor size has been shown to be associated with response to immunotherapies [52], but several scenarios, from surveillance to evasion, are known to occur [31, 53, 54]. Is genetic instability related to the polymorphic nature of immunotherapy prognosis?

From Fig. 4a we know that, in conditions of low genetic instability, the large tumor equilibrium is globally asymptotically stable (GAS), and insufficient presentation of antigens implies that even small tumors can evade immune surveillance in the absence of a strong innate response through NK cells or macrophages. This could be the case of both initial microsatellite-stable malignancies or clones that have evolved low antigenicity through genome editing [45].

Increases in genetic instability result in a phase transition that creates a micro-tumor attractor (Fig. 4b-c). This state has been previously related to dormancy, where the adaptive immune system is able to control cancer growth [31]. However, the large cancer attractor is still present, and local asymptotic stability ensures that tumor sizes within its basin of attraction will stil grow towards it. The implications are revelant to therapy: small tumors of medium antigenicity can be controled, but large tumors will still grow towards larger disease. This result is consistent with the notion that therapy reducing tumor mass is often effective prior to immunotherapy [20, 55].

The second transition, consistent with experiments of immune surveillance after Mismatch-Repair Knockout [17], indicates the disappearance of the large cancer attractor (Fig. 4d). This implies that highly immunogenic tumors will always elicit a sufficiently effective immune response that will drive them towards microtumor control [31], no matter their initial size. However, the fact that there is no complete remission implies that evolutionary pressures still act on the remaining rogue population, and the small clone can eventually evolve immune evasion [45].

Mutagenic therapy remains a relevant actor on the cancer-immune ecology. However, without the cooperative effects of an innate response, through the constant recognition rate *ρ*_*NK*_, or the buffering of immune migration *m*, the cancer-free equilibrium is only stable at very high genetic instability levels that do not seem attainable through mutagenic agents. What are the cooperative dynamics of genetic instability with these immune agents?

### Effects of modulating immune migration and the innate response

Beyond the relevance of genetic instability as a driver of tumor antigenicity, the fact that the cancer free attractor becomes stable at very high mutational levels above 10^−4^ mutations per gene and division (at least for the data on adaptive immunity from [20]) implies that further considerations on therapy need to be taken into account. The overall condition for total disease eradication is

12 $$ \frac{r(\mu)}{\rho_{NK} + \rho_{T}(\mu)} < \frac{m\delta}{d}.  $$

If genetic instability alone does not suffice to fulfill this condition, what other therapeutic schemes are of relevance to our model? A first notion lies on understanding how does *μ* alter the minimal innate recognition *ρ*_*NK*_ necessary for complete disease remission, as defined by the condition

13 $$ \rho_{NK}^{*} > \frac{r(\mu)d}{m\delta} - \rho(\mu)  $$

For microsatellite stable tumors with *μ*<<10^−5^, the necessary recruitment rate of NK cells is within the 10^−1^*day*−1 range, an order of magnitude larger than that measured in [27]. However, increasing genetic instability decreases $\rho _{NK}^{*}$ in a quasi-linear way, so that after a possible MMR knockout, a recruitment rate within 10^−2^*day*^−1^ would suffice for cancer clearance, indicating the possibility of a combination therapy enhancing both mutagenesis and NK cell activation [28].

Together with the role of innate immunity, another key observation is considering the rate of immune migration (*m*) as a measure of immune activation. The necessary flow of immune cells to the tumor to achieve complete remission is

14 $$ m^{*}> \frac{r(\mu)d}{\delta(\rho_{NK} + \rho_{T}(\mu))}  $$

Interestingly enough, the migration rate necessary for cancer clearance does not decay linearly with genome instability, as for $\rho _{NK}^{*}$, but in an exponential way, meaning that increases in genetic instability within the MMR knockout range rapidly decrease the condition for immune migration rate (Fig. 4f). This indicates a strong synergy between mutagenesis and immune activation therapies such as Adoptive Cell Therapy (ACT) [56], consistent with recent discussion on combination therapies [7, 19].

Moreover, by picturing the bifurcation diagram in standard *μ* and *m* regions as described in the Methods section (Fig. 4e), it is interesting to see how the first transition towards microtumor creation, $\mu ^{*}_{1}$, has a weak dependency on *m*, since the appearance of the intermediate attractors depends mostly on the denominator of nullcline 2 becoming null, so that *E*_2_(*c*) diverges at

15 $$ \delta_{E}c + d - ((\rho_{NK} + \rho_{T}(\mu)) c/(g + c))=0,  $$

which is not a function of *m*. On the other hand, the transition to disappearance of the large-cancer attractor does depend on *m*, since *m* affects the width of *E*_2_(*c*), so that for higher *m* values *E*_2_(*c*) will go faster towards infinity and not cross *E*_1_(*c*). However, it seems intuitive from Fig. 4f that the role of genetic instability in increasing neoantigen production might be crucial even in the presence of high immune activation.

Mathematical work previous to our instability-driven model developed interesting considerations on derivation of cancer vaccines (see e.g. [57]), and introduced time dependent treatments [58] or time-delays in the immune response [59] based on the immune migration parameter, despite mathematical considerations remained somehow distant from clinical immunology and not many of the described behaviors after mathematically designed therapies have been observed in vivo [22].

Recent research has highlighted the importance of genetic instability as a marker for good prognosis in immune checkpoint inhibition therapies [14–16]. Its role in neoantigen production is acknowledged as crucial [10]. Our results describing *μ* as another driver towards surveillance complementing *m* and *ρ*_*NK*_ reinforce the relevance of genetic instability in the tumor-immune dynamics, further supporting the possibility of increasing tumor immunogeneicity by promoting T cell antigen presentation [7, 9].

## Discussion

In the present work we have studied a minimal mathematical scenario describing how genetic instability, by means of enhancing tumor adaptation along with neoantigen production and immune recognition, can trigger sharp transitions towards tumor control and eradication.

Starting from basic considerations, we have asked ourselves about the ecological interactions between malignant cells and, in particular, effector immune cells able to respond after neoantigen recognition. Specifically, we consider how genetic instability, here as a mutation probability, shapes tumor adaptability and immune response.

Interestingly, genetic instability governs the possible outcomes of the system. Increasing mutational levels drive the system across two phase transitions. In the first one, two attractors are created involving smaller tumors coexisting with a larger population of T cells. This state has been characterized as a controlled, but not totally eliminated microtumor [30, 31]. The second transition accounts for the disappearence of the cancer-wins scenario, so that only solutions of immune control are present at large genetic instability levels.

Recent advances in the field of cancer immunology have proven that genetic instability is a key ingredient of the immune response [14–16], and particular research claims immune surveillance after MMR knockout follows from this causal relation between high mutational loads and neoepitope production [17]. In the context of this research, our model provides a conceptual and numerical description for how a transition between cancer growth and arrest can follow only from damaging DNA repair mechanisms. More generally, the fact that microsatellite instability levels govern transitions separating cancer growth from immune surveillance might be indicative of why highly unstable tumors are better respondants to immunotherapy [10]. Furthermore, we have used available data to calibrate the model parameters and to construct the immune recognition function. Using this information, we consistently explain phase transitions happening at microsatellite instability levels that resemble those of MMR knockout. However, even if these transitions could exist in the laboratory, we have discussed further aspects that need to be accounted when dealing with increasing tumor immunogeneicity through mutagenesis [49, 50].

We have also studied the roles of *ρ*_*NK*_, the recruitment of NK cells, and *m*, a parameter refering to immune migration or an eventual immune therapy. The model indicates a cooperative effect between therapies affecting mutagenesis together with NK or migration buffering. The strength of this cooperative effect is linear for genetic instability and innate immune cell recruitment, but the model also predicts that, when an innate response and T cell recognition alone cannot control tumor growth, cross-therapies modulating both *m* and *μ* might be exponentially effective in driving the tumor-immune interaction into a state of total disease eradication, thus indicating a mathematical validation for recent insight into combined immunotherapies [7]. We further suggest that the relevance of *m* in producing transitions to tumor arrest is low, while minor increases in genetic instability seem much more effective against large tumors. This indicates that cross therapies inducing DNA damage prior to immunotherapy might drive tumors to neoantigen-rich states [18, 19] before immune editing processes enter at play [45, 60]. We therefore postulate a possible mathematical description of recent discussions for novel perspectives on combination immunotherapy [7].

All the previous conclusions stem from a very minimal mathematical model, whereas the immune system is known to be complex [45, 61] Additionally, other interactions between immunotherapies and conventional therapies need to be taken into account [19]. In particular, several cooperative mechanisms between immune populations might play a role in non-antigenic T cell activation [27]. Further research should consider the possible nonlinear dynamics stemming from T cell sensitization after cancer-NK cellular interactions.

Finally, as a result from the lack of heterogeneity, our model does not yet capture immune editing, a phenomenom at the core of immunotherapy failure, in which the tumor might develope immune resistance by means of either buffering the growth of immunosilent cells or editing its genome to express fewer neoantigens [60]. Within this view, current research claims that tumor mutational burden might not be a sufficient biomarker [46, 50]. In the presence of an effective immune response, antigenic subclones can be negatively selected, giving rise to immuno-silent tumors despite its possibly high mutational load. Together with immune editing, recent studies highlight heterogeneity itself as a source for failure of the immune response [49, 51] as it directly affects the spatial and clonal distribution of neoantigens. Further modeling of the tumor-immune ecology could benefit from considering heterogeneous populations where antigen frecuencies are taken into account. Despite these considerations, our results on the cooperative roles of *m* and *μ* indicate that damage on DNA repair mechanisms prior to checkpoint blockade could render tumors immunogenic before a reactivated immune system pressures towards editing. Using an evolutionary framework such as adaptive dynamics [37], future work might help to characterize in which regimes do cancer subclones evade immune surveillance through evolving their neoantigen landscape [62].

## Conclusions

This work provides a first effort towards modeling the double-edged effect of genetic instability in both cancer adaptation and immune surveillance with the goal of understanding the specific role of mutational load as a driver of immune attack. Two main results stem from the model. First, transitions towards tumor control follow from increases in mutational levels similar to those after MMR knockout. Second, genetic instability and immune activation have a cooperative effect in driving tumor elimination, indicating that combination therapies enhancing both might be key in the future.

## Data Availability

Not applicable
